# Fibrinogen-like protein 1 (FGL1): the next immune checkpoint target

**DOI:** 10.1186/s13045-021-01161-8

**Published:** 2021-09-15

**Authors:** Wenjing Qian, Mingfang Zhao, Ruoyu Wang, Heming Li

**Affiliations:** 1 Department of Oncology, Affiliated Zhongshan Hospital of Dalian University, No. 6 Jiefang Street, Dalian, Liaoning 110006 People’s Republic of China; 2 The Key Laboratory of Biomarker High Throughput Screening and Target Translation of Breast and Gastrointestinal Tumor, Dalian, 116001 People’s Republic of China; 3 Department of Medical Oncology, the First Hospital of China Medical University, No.155 Nanjingbei Road, Shenyang, Liaoning 110001 People’s Republic of China

**Keywords:** FGL1, LAG-3, Biomarker, Immune resistance, Immune checkpoint blockade

## Abstract

Immune checkpoint therapy has achieved significant efficacy by blocking inhibitory pathways to release the function of T lymphocytes. In the clinic, anti-programmed cell death protein 1/programmed cell death ligand 1 (PD-1/PD-L1) monoclonal antibodies (mAbs) have progressed to first-line monotherapies in certain tumor types. However, the efficacy of anti-PD-1/PD-L1 mAbs is still limited due to toxic side effects and de novo or adaptive resistance. Moreover, other immune checkpoint target and biomarkers for therapeutic response prediction are still lacking; as a biomarker, the PD-L1 (CD274, B7-H1) expression level is not as accurate as required. Hence, it is necessary to seek more representative predictive molecules and potential target molecules for immune checkpoint therapy. Fibrinogen-like protein 1 (FGL1) is a proliferation- and metabolism-related protein secreted by the liver. Multiple studies have confirmed that FGL1 is a newly emerging checkpoint ligand of lymphocyte activation gene 3 (LAG3), emphasizing the potential of targeting FGL1/LAG3 as the next generation of immune checkpoint therapy. In this review, we summarize the substantial regulation mechanisms of FGL1 in physiological and pathological conditions, especially tumor epithelial to mesenchymal transition, immune escape and immune checkpoint blockade resistance, to provide insights for targeting FGL1 in cancer treatment.

## Background

Immune checkpoints (ICs) are essential in modulating the immune response and mediate T cell dysfunction in autoimmunity and inflammation [1–5]. However, these inhibitory pathways can be educated by tumor cells and promote tumor immune escape [6–8]. Recent cancer immune checkpoint blockade therapies have aimed to reverse such T cell exhaustion by targeting immune checkpoints “to release the brakes”, which allows cytotoxic T cells to attack tumor cells [4, 9–11]. In recent years, targeted cytotoxic T lymphocyte-associated antigen-4 (CTLA-4) and/or PD-1/PD-L1 therapies have been used in the treatment of clinically advanced tumors and have achieved high rates of objective remission [12–14]. Unfortunately, several cancer patients receiving PD-1/PD-L1 mAb therapy have exhibited pseudoprogression (PP) [15, 16] or hyperprogression (HP) [17–20]. Although PP is considered a rare phenomenon with varying incidence rates (approximately 1–10%) in different tumors using different assessment criteria (Response Evaluation Criteria in Solid Tumors (RECIST) and immune-related response criteria (irRC)), anti-PD-1/PD-L1 therapy still needs to be treated with caution to achieve higher survival benefits [15]. Additionally, previous studies reported that 9% of 131 evaluable patients were considered to have HP (defined as a twofold increase in the tumor growth rate between the reference and experimental periods) [17]. In addition, patients treated with nivolumab (a humanized mAb against PD-1) were found to be at a higher risk of developing side effects, such as interstitial pneumonia and colitis [21–25]. Most importantly, it has been confirmed that only 20% of patients benefit from immune checkpoint blockade therapy in clinical studies [26], with the rest of the patients showing primary or adaptive drug resistance to varying degrees [27–37]. Therefore, the efficacy of immunotherapy is not satisfactory. Thus, attention has turned to other effective immune checkpoint pathways in further studies [12, 38–41].

Recent studies have focused on one of the most promising immune checkpoints. LAG3 (CD223) is an inhibitory receptor expressed mainly on the surface of T lymphocytes [26, 42, 43]. The binding of LAG3 with its ligands delivers negative signals to activated T cells, preventing immune-mediated tissue damage [44]. Similar to PD-1/PD-L1 [5, 45, 46], LAG3 has an inhibitory function based on interactions with its ligands, which include major histocompatibility complex II (MHC II) [47, 48], galectin-3 [49], liver sinusoidal endothelial cell lectin (LSECtin) and FGL1 [12, 50]. FGL1, also known as liver fibrinogen-related gene-1 (LFIRE-1)/Hepassocin (HPS) or hepatocyte-derived fibrinogen-related protein-1 (HFREP-1) [51–53], is a proliferation- and metabolism-related factor secreted by the liver [54–56]. It has recently emerged as a novel ligand of LAG3 beyond the classic ligand MHC II and can bind with LAG3 to form a new immune checkpoint pathway independent of PD-1/PD-L1, which results in T cell depletion and subsequent dysfunction, as well as tumor cell escape from immune surveillance [50, 57]. Apart from its relatively high expression in the liver and pancreas, FGL1 is upregulated in tumor tissues (including lung, prostate, melanoma, colorectal, breast and brain tumors) based on several datasets [50]. Hence, FGL1 has potential as another immune checkpoint target in clinical practice, especially in targeted therapy for non-small cell lung cancer (NSCLC) [58–65]. In this review, we summarize the function and molecular mechanism of FGL1 in the regulation of cancer development and metastasis and provide promising applications in therapeutic strategies for malignant tumor treatment.

## FGL1 biological function and regulation of expression

### Structure and distribution of FGL1

**Fig. 1:**
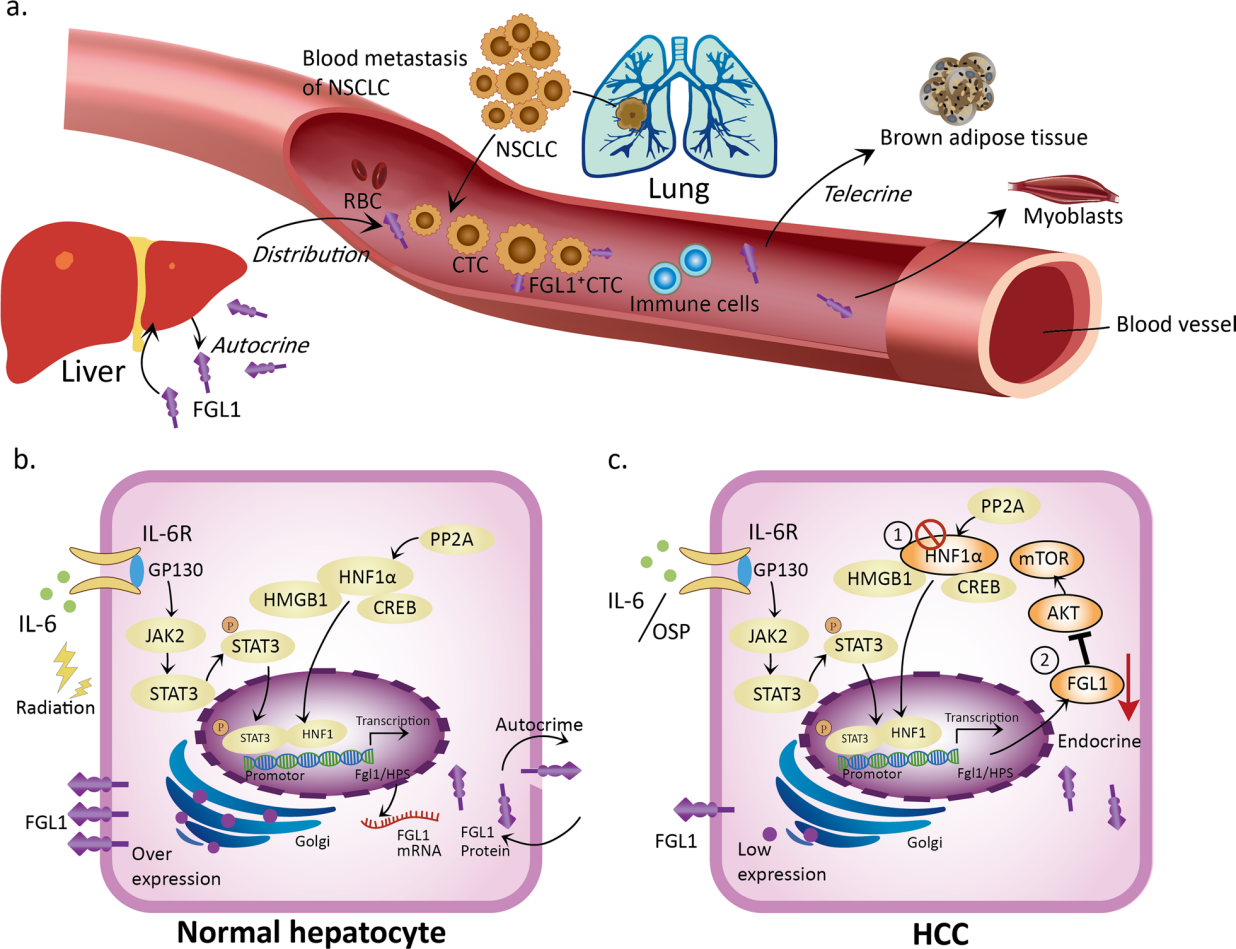
The mechanism by which hepatocytes express and secrete FGL1 under physiological and pathological conditions. **a** FGL1 has two origins, with the liver being the classic source. FGL1 secreted by the liver can be distributed in adipose tissue and circulate in the blood. Tumor tissues, such as cancerous lung tissues (e.g., NSCLC), were recently identified as a new source of FGL1; FGL1 can also be detected on the surface of CTCs in NSCLC patients. FGL1 acts on both adipocytes and myoblasts via exerting telecrine effects. **b** The mechanism of normal hepatocyte expression and secretion of FGL1 under physiological conditions: In normal hepatocytes, the JAK2-STAT3 pathway is activated by either the inflammatory factor IL-6 or radiation. HNF1α forms a complex by binding to the HMGB1 and CREB proteins in the cytoplasm. Then, it enters the nucleus and binds to the HPS promoter through pSTAT3, thereby upregulating FGL1 transcription. The overexpressed FGL1 protein is distributed via an autocrine process. **c** The mechanism of low FGL1 expression in HCC: The IL-6-JAK2-STAT3 pathway, which induces FGL1 expression, can be blocked by oxysophocarpine (OSP). Deletion of HNF1α and inhibition of the AKT-mTOR pathway downregulate FGL1 via an endocrine mechanism

FGL1 is located on human chromosome 8 (8p22-21.3) and is a 68-KD protein comprised of a disulfide bond-linked homodimer [66, 67]. Its carboxyl terminus contains the β and γ subunits, which are highly homologous to fibrinogen but irrelevant to coagulation-related binding sites [50]. Under normal physiological conditions, FGL1 is secreted mainly by hepatocytes in the liver (some of which may also exist in the pancreas) [50]. It is now clear that FGL1 is the product of hepatocyte regeneration and participates in hepatocyte mitosis and liver energy utilization (including lipid metabolism and blood glucose regulation) [54, 68, 69]. Apart from the above functions, FGL1 can also be detected in the plasma as an acute reactant [70], implying that FGL1 secreted by the liver acts not only on hepatocytes (autocrine) but also on other tissues, such as muscle and brown adipose tissues (telecrine) (Fig. 1a) [56, 68]*.* Under stimulation by metabolic factors (hyperglycemia, hyperlipidemia, hormones, etc.), the liver secretes FGL1 and participates in the blood circulation [52, 69, 71]. This function of FGL1 acts on brown adipose tissue [54, 56], regulates body productivity and maintains body temperature. Moreover, FGL1 also acts on muscle tissue and affects the sensitivity of myoblasts to insulin [52, 68].

### Expression, regulation and function of FGL1 in benign disease

Given that FGL1 is a physiological secretory factor of the liver, its expression can be regarded as a self-protective mechanism acting against exogenous injury or stimulation. FGL1 expression is regulated by radiation-induced lung, liver and stomach injury [71–73]. In addition, factors released by hepatectomy and postoperative inflammation (interleukin (IL)-6 and transforming growth factor (TGF)-β) can also affect the expression of FGL1 [70, 73–77]. Under such exogenous induction, normal hepatocytes regulate FGL1 transcription via signal transducer and activator of transcription 3 (STAT3) and hepatocyte nuclear factor-1α (HNF-1α) (Fig. 1b) [78–83]. FGL1 expression can also be regulated in chronic medical diseases, such as hyperlipidemia, insulin resistance, and hyperglycemia crisis. Hitherto, there have been studies on the mechanism of the FGL1-mediated regulation of substance metabolism in chronic diseases (Table 1). In clinical practice, changes in the FGL1 expression level can be specifically observed in type 2 diabetes patients with non-alcoholic liver disease [52]. Recently, plasma tests in these patients have found that FGL1 is upregulated when insulin resistance occurs. The high concentration of FGL1 in the plasma acts not only on itself but also on both adipocytes and myoblasts (Fig. 1a). In a hyperlipidemic model, palmitate-treated primary hepatocytes regulated the expression of FGL1 through CCAAT/enhancer-binding protein β (C/EBPβ)-mediated transcription, and the expression level of FGL1 in the liver of mice fed a high-fat diet (HFD) was also increased [68]. This high level of FGL1 could act on C2C12 cells (myoblasts) and generate insulin resistance through the phosphorylated JNK pathway [68]. In addition, in patients with type 2 diabetes, FGL1 induces insulin resistance in HepG2 cells via the hepatocyte-dependent extracellular regulated protein kinase (ERK)1/2 pathway [52]. Furthermore, in adipocytes, extracellular FGL1 induces adipogenesis through the canonical ERK1/2-C/EBPβ-peroxisome proliferator-activated receptor (PPAR) γ pathway [56].

**Table 1:** Regulation of FGL1 expression in association with substance metabolism

Stimulating factor	Induced cell type	Signaling pathway	Phenotype	References
FGL1	3T3-L1 (adipocyte)	ERK1/2-C/EBPβ-PPARγ	FGL1-induced adipogenesis	[56]
	HepG2	ERK1/2	Impair insulin sensitivity	[52]
Hyperglycemia	HepG2	ERK1/2-NRF2-SOD1	Enhance antioxidative stress	[69]
		STAT3/HNF-1	Upregulate FGL1 expression	
Hyperlipidemia (Palmitate)	Primary hepatocyte	C/EBPβ	Upregulate FGL1 expression	[68]
FGL1	C2C12 (myoblast)	JNK-p-JNK	Impair insulin sensitivity	
Hepatectomy/IL-6	Hepatocyte	HNF-1/STAT3/AP-1	Activate the FGL1 and IGFBP-1 genes; restore metabolic homeostasis	[74, 78]

AP-1: activating protein-1; C/EBPβ: CCAAT/enhancer-binding protein β; ERK1/2: extracellular regulated protein kinases 1/2; HNF-1: hepatocyte nuclear factor-1; IGFBP-1: insulin-like growth factor binding protein 1; JNK: Jun N-terminal kinase; NRF2: nuclear factor erythroid-2-related factor 2; PPARγ: peroxisome proliferator-activated receptor gamma; SOD1: superoxide dismutase 1; STAT3: signal transducer and activator of transcription 3

Apart from protecting hepatocytes (promoting regeneration and apoptosis) and regulating metabolism, FGL1 expression can also be used as a marker of some benign diseases (autoimmunity, infectious diseases, acute inflammation, etc.). First, the value of FGL1 can predict the activity of rheumatoid arthritis (RA; moderate/high: 91.46%; remission/low: 80.77%) and the severity of dengue fever [84, 85]. Second, the fluctuation in FGL1 expression in vivo can also affect the progression of obesity and malaria parasite infection [56, 86]. These above studies reveal that FGL1 plays important roles and acts as a potential biomarker in several common benign diseases.

## Role of FGL1 in cancer development

### FGL1 is upregulated in solid tumors

At present, studies on the regulation of FGL1 expression are not limited to the exploration of its physiological and pathological functions [87]. The expression of FGL1 in human solid tumors is different from that in paracancerous tissues. According to the bioGPS tissue microarray database and a proteomic analysis, both the mRNA and protein expression levels of FGL1 are mainly confined to the normal liver and pancreas in humans. A meta-analysis of Oncomine datasets showed that FGL1 expression was upregulated in lung, prostate, melanoma, colorectal, breast cancer and brain tumors but downregulated in pancreatic, breast, liver and head and neck cancers. Among these changes, the trends observed in lung, prostate and liver tumors were consistent with those in The Cancer Genome Atlas (TCGA) datasets, while FGL1 was significantly upregulated in the lung adenocarcinoma cancer map (FGL1 ranked 38th among the top 200 highly expressed genes in lung cancer). In addition, multiple quantitative immunofluorescence (QIF) staining was performed on a tissue microarray of 275 NSCLC samples to detect FGL1 protein expression in cells and tissues (Table 2). The data showed that the FGL1 protein was expressed in local pan-keratin-positive tumor cells but the stromal compartment exhibited almost no expression. These above results indicate that the expression of FGL1 is upregulated in human tumors, as represented by NSCLC [50]. Moreover, FGL1 is downregulated in hepatocellular carcinoma (HCC) in TCGA datasets. This result was also confirmed in a recent study showing that the expression of FGL1 could be downregulated due to the deletion of HNF1α in HCC [51, 67, 78] (Fig. 1c). Hence, FGL1 is released at lower levels by HCC tumor tissues but at higher levels in other solid tumors.

**Table 2:** Summary of the FGL1 detection in various human cancers as determined by a clinical approach

No	Year	Tumor type	Numbers/samples	FGL1 detection methods	Conclusions	References
1	2020	BC	*N* = 47, primary tumor tissue; *N* = 82, peripheral blood	IHC; flow cytometry	FGL1 was present and tended to be expressed at higher levels in stage III cancer cells than in stage I or II cancer cells	[163]
2	2020	HCC	*N* = 143, primary tumor tissue	Multiplex IF	High FGL1 expression was negatively associated with PD-L1 expression and the CD8^+^ T cell density but positively associated with high LAG3^+^ T cell density	[165]
3	2019	LUAD	*N* = 30, primary tumor tissue	IHC	Low FGL1 expression contributed to EMT and angiogenesis in LKB1-low LUAD tissue samples	[93]
4	2019	NSCLC	*N* = 275, plasma	Multiplex QIF	FGL1 was shown to exhibit a relatively high expression level in tumor cells compared with the stromal distribution and paired normal tissues; ~ 15% NSCLC patients showed elevated expression, implying a worse 5-year OS rate	[50]
			*N* = 74, plasma	ELISA	Higher plasma FGL1 levels were detected in NSCLC patients than in healthy donors; the plasma FGL1 levels in NSCLC patients were not associated with tumor metastasis or liver injury	
			*N* = 18, plasma	ELISA	Higher plasma FGL1 levels were associated with worse OS in NSCLC patients treated with anti-PD-1/PD-L1 therapy	
5	2019	MM	*N* = 21, plasma	ELISA	Higher plasma FGL1 levels were associated with worse OS in metastatic melanoma patients treated with anti-PD-1/PD-L1 therapy	
6	2019	GC	*N* = 50, primary tumor tissue	qPCR and WB	Both the mRNA and protein levels of FGL1 were obviously higher in GC tissues than in normal tissues (*P* < 0.001); high FGL1 expression was related to a poor prognosis (*P* < 0.01); the FGL1 expression, pathological stage and histological grade were positively associated with the OS of GC patients (*P* < 0.05)	[94]

BC: breast cancer; ELISA: sandwich enzyme-linked immunosorbent assay; GC: gastric cancer; IF: immunofluorescence staining; IHC: immunohistochemical staining; QIF: quantitative immunofluorescence staining; LUAD: lung adenocarcinoma; MM: melanoma; OS: overall survival; qPCR: quantitative PCR; WB: western blot

### FGL1 mediates EMT process in tumors

**Fig. 2:**
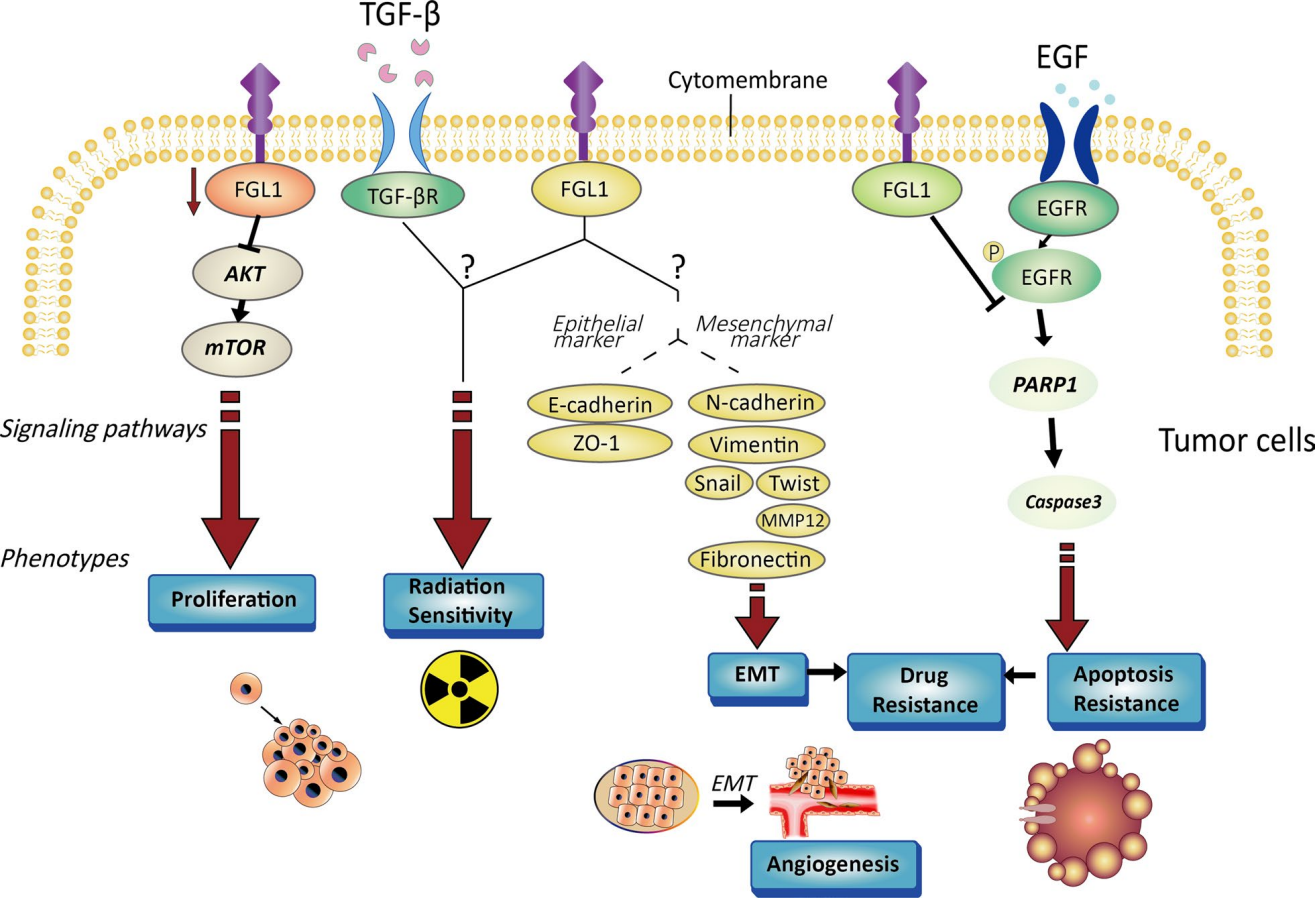
Characterization of tumor cells regulated by FGL1. FGL1, which is expressed in tumor cells, regulates cell proliferation, apoptosis, EMT, drug resistance and radiation sensitivity. FGL1 regulates downstream signaling pathways related to cell proliferation by modulating the AKT-mTOR pathway. FGL1 mediates EMT by regulating epithelial markers (E-cadherin and ZO-1) and mesenchymal markers (N-cadherin, vimentin, Snail, Twist, MMP12 and fibronectin). In addition, FGL1 mediates PARP1-caspase apoptotic pathways by affecting EGFR phosphorylation. Radiation sensitivity may be pertinent to TGFβ signaling. The processes of EMT and tumor cell apoptosis are closely related to drug resistance

The process of tumor progression is accompanied by changes in angiogenesis, invasion and migration [88]. Tumor epithelial to mesenchymal transition (EMT) is not only reflected in changes in cell morphology but also closely related to the behaviors of tumor cell invasion and migration [89–92]. Previous preclinical studies have confirmed the correlation between FGL1 and the tumor EMT process in lung and gastric cancers [93, 94]. First, following FGL1 silencing in liver kinase B1 (LKB1)-mutant lung adenocarcinoma cells (A549 and H157 cells), E-cadherin was shown to be downregulated, while N-cadherin and vimentin were upregulated [93, 95, 96]. However, another opposite conclusion was also reached: E-cadherin was upregulated and the expression of N-cadherin and vimentin was suppressed when FGL1 was knocked out in SGC-7901 gastric cancer cells [94, 97–101]. Hence, the relationship between FGL1 and EMT progression in tumor cells still needs to be further investigated. In addition, a correlation between FGL1 and the EMT process has also been reported in a pulmonary fibrosis model, which is distinct from tumor models. Knockout of FGL1 impeded the processes of radiation- or TGF-β-induced EMT [73, 89]. After silencing the FGL1 gene in L132 cells, EMT markers (increased: Snail [102], Twist [103], MMP12 and fibronectin [104–106]; decreased: ZO-1 [107, 108]) changed at both the mRNA and protein levels. These above studies demonstrate that there is a correlation between FGL1 and the EMT process in tumors but the conclusions are not consistent. In the presence of FGL1, interstitial markers are downregulated, confirming that FGL1 modulates EMT under radiation or inflammatory induction [109]. This effect may be closely related to the protective effect of FGL1 in response to injury stimulation. As tumor EMT is tightly associated with prognosis and drug resistance [110–113], the specific mechanism by which FGL1 regulates tumor EMT needs to be clarified (Fig. 2).

### FGL1 regulates other tumor characteristics

Apart from mediating the EMT process, FGL1 is also involved in tumor proliferation, apoptosis, radiation and drug sensitivity [71, 72, 114–116]. Tumor growth is closely correlated with cell proliferation and apoptosis. The clonal proliferation ability of PC9/GR cells was evaluated in a study on targeted drug resistance in NSCLC, and the data showed that the proliferation of PC9/GR cells decreased significantly after FGL1 silencing [114]. Knockout of FGL1 could significantly dampen the proliferation of SGC-7901 cells [94]. It has been reported that the activation of mTOR is tightly related to the proliferation of HCC cells [117]. As an inhibitor of HCC, FGL1 regulates the occurrence of HCC mainly by inhibiting AKT and downstream pathways, indicating that AKT-mTOR is an important pathway downstream of FGL1 involved in regulating cell proliferation [67, 118]. On the other hand, in the mechanism of FGL1-mediated gefitinib resistance, FGL1 was found to regulate apoptosis through the PARP1/caspase-3 pathway [114], which also confirmed that FGL1 is pertinent to the sensitivity of tumor cells to targeted EGFR-tyrosine kinase inhibitors (TKIs) (Fig. 2). A recent study on FGL1-mediated targeted therapy resistance have also been reported in HCC [116]. In addition, although FGL1 is a mediator of radiation injury [71–73], the correlation between FGL1 and radiation sensitivity needs to be confirmed by additional studies.

## FGL1 and tumor immune escape

In the tumor microenvironment (TME), some tumor and regulatory immune cells, such as regulatory T cells (Tregs) [119–122], myeloid-derived suppressor cells (MDSCs) [123–128], tumor-associated macrophages (TAMs) [129–131], immature dendritic cells (iDCs) [132, 133] have the ability to generate inhibitory molecules (like TIM3 [134–136], TIGIT [137, 138] and VISTA [139]), which can bind receptors on immune cells (NK cells, lymphocytes, etc.), reducing the toxicity of immune responses and thus contributing to immune escape. These molecules play important roles in antitumor efficacy and outcome prediction. The most representative immune checkpoint targets are PD-1 [4, 11, 140, 141] and PD-L1 [82, 123, 128, 142–145]. More recently, Chen and his coworkers have elucidated that the FGL1/LAG3 pathway is another encouraging immune checkpoint pathway that plays a crucial role in the immune escape mechanism, similar to PD-1/PD-L1, and potentially mediates resistance to anti-PD-1/PD-L1 therapy. Thus, as a LAG3 ligand, FGL1 will be another promising biomarker with predictive value for PD-1/PD-L1 resistance [50].

### FGL1 is a major immune inhibitory ligand of LAG3

**Fig. 3:**
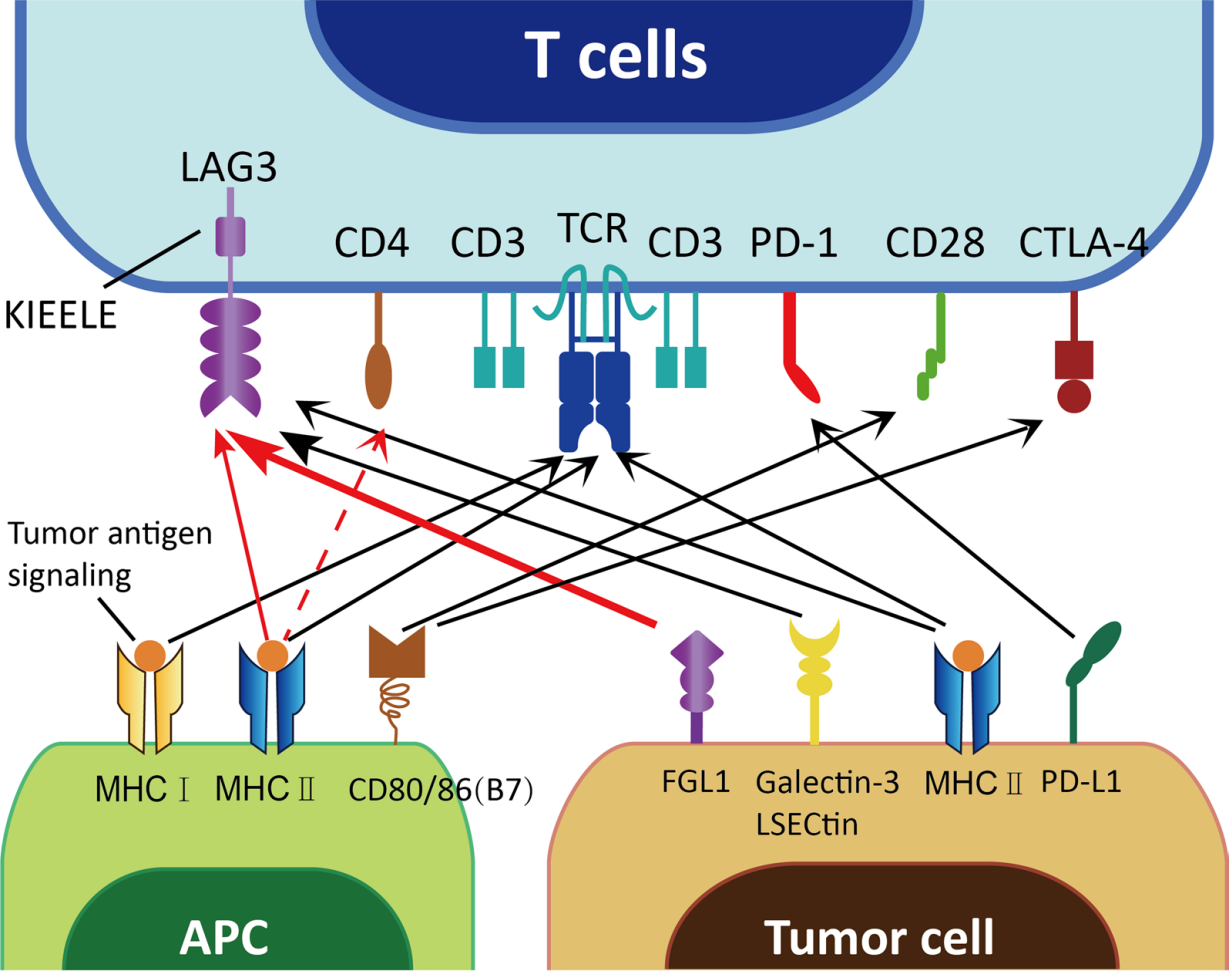
Classic and novel immune checkpoints between tumor cells and immune cells. The traditional marker PD-L1 (B7-H1/CD274), expressed on the surface of tumor cells, binds to PD-1 on the T cell surface, while the newly identified molecule FGL1 binds to another inhibitory receptor, LAG3, which has other ligands, such as MHC II, galectin-3 and LSECtin (degree of affinity: thick line > thin line > dashed line). In addition, CD80 and CD86 (B7) on the surface of APCs bind with CD28 or CTLA-4 to provide auxiliary stimulatory and suppressive signals, respectively

FGL1 was first identified as a new high-affinity ligand for the inhibitory receptor LAG3 by Dr. Chen’s team in 2019 [50]. LAG3 is a type I transmembrane protein that exists mainly on the surface of activated T cells (including CD4^+^ and CD8^+^ T cells and Tregs) [146–148], NK cells [149], plasmacytoid dendritic cells (pDCs), and so on. The extracellular domain of LAG3 is 20% similar to that of CD4 [12], which determines the high affinity between LAG3 and MHC II. LAG3 interacts with MHC II and generates a negative signal (blocking the TCR activation signal) to restrict T helper 1 (Th1) cell activation, proliferation and secretion [150, 151]. Conversely, activated T cells expressing LAG3 stimulate the production of cytokines (tumor necrosis factor (TNF)-α and IL-12) in antigen-presenting cells (APCs) (DCs and monocytes) and induce their maturation and activation [152]. Notably, researchers found that junction peptides between the D4 transmembrane region and transmembrane region could be cleaved to generate soluble LAG3 (s-LAG3) [12, 153, 154], which could activate thymic epithelial cells and increase CD4^+^ T cell levels through the transduction of MHC II signals (Fig. 3).

In some subsequent studies, it was confirmed that MHC II is not the only ligand of LAG3 [43]. Most recently, evidence has demonstrated that FGL1 is another type of high-affinity immunosuppressive ligand that binds to LAG3. A number of basic research studies have revealed that there is a high-affinity interaction between LAG3 and FGL1 [13]. In the Genome-Scale Receptor Array (GSRA) system, FGL1 was the main binding protein of LAG3-Ig, and the interaction between them was stable, specific and conserved across species. A further domain deletion study showed that the fibrinogen-like domain (FD) in FGL1 and the D1-D2 domain in LAG3 are involved in the MHC II-independent interaction between FGL1 and LAG3. Based on in vivo experiments, loss of the FGL1/LAG3 interaction could be achieved by gene knockout or antibody blockade, and antitumor immunity could be promoted by stimulating tumor-infiltrating lymphocyte (TIL) activation and expansion in the TME [50].

### Synergistic inhibition of T cells with PD-1 and FGL1 as a LAG3 ligand

Although the specific mechanism by which FGL1/LAG3 modulates T cell functions remains unclear, the synergistic inhibitory effect of FGL1 blockade in conjunction with anti-PD-1 therapy has been confirmed in animal models [50]. It has been indicated that blocking FGL1 can cooperate with anti-PD-1/PD-L1 therapy to suppress the effect of PD-1/PD-L1 signaling, which is based on previous basic research studies on the synergistic inhibitory activities [146, 153, 155–157]. The synergistic inhibitory effect of LAG3 and PD-1 on T cells has been confirmed in a variety of tumors, including NSCLC [158], melanoma [159], renal cell carcinoma (RCC) [160, 161], head and neck cancer [6], and breast cancer [162, 163]. Among these diseases, melanoma shows a close relationship between the coexpression of the immune checkpoint molecules LAG3 and PD-1 and the expression of CD163 and density of TAMs [129, 164]. These preclinical results have paved the way for combinatorial blockade of PD-1 and LAG3 in clinical trials [165]. Most importantly, FGL1 will be a potential biomarker for predicting the outcome of PD-1/PD-L1 blockade therapy, since high plasma FGL1 levels were reported to be significantly correlated with a worse therapeutic response to anti-PD-1/PD-L1 therapy in NSCLC and melanoma patients [50, 166]. Therefore, FGL1 can be identified as a next-generation cancer immunotherapy target capable of a functional interaction with the LAG3 pathway and synergistic inhibition of T cells with PD-1.

### FGL1/LAG3 is an immunosuppressive pathway independent of PD-1/PD-L1

**Fig. 4:**
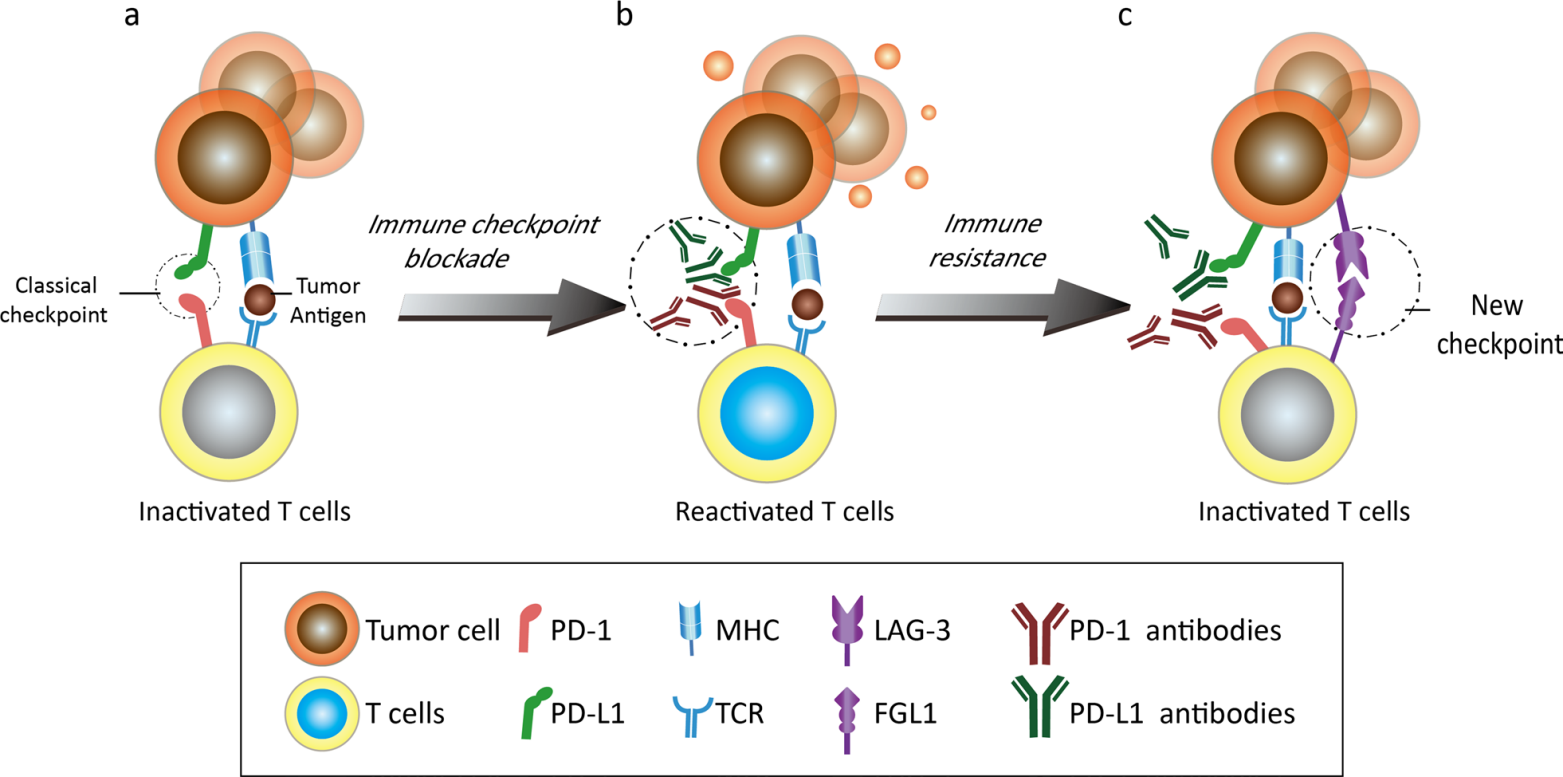
Schematic representation of the mechanism by which tumor cells become resistant to anti-PD-1/PD-L1 antibodies via the FGL1/LAG3 pathway. **a** The PD-1/PD-L1 pathway inhibits the toxic effects of T cells on tumor cells. **b** The addition of anti-PD-1/PD-L1 antibodies restores the toxic effect of T cells on tumor cells. **c** The FGL1/LAG3 pathway inactivates T cells, and tumor cells become resistant to anti-PD-1/PD-L1 antibodies once again

A series of immunological animal experiments have confirmed the synergistic effect of the inhibitory receptors LAG3 and PD-1 on T cells [146, 157]. However, the FGL1/LAG3 pathway also plays an immunosuppressive role independent of the PD-1/PD-L1 pathway. It is well known that the PD-1/PD-L1 inhibitory pathway produces exhausted T cells (Fig. 4a). Anti-PD-1/PD-L1 antibodies can block this immune brake and release the antitumor activity of T cells (Fig. 4b). However, due to the existence of another inhibitory receptor, LAG3, on the T cell surface, another newly characterized immune checkpoint is generated when LAG3 interacts with its ligand FGL1 (Fig. 4c). It has been reported that blocking LAG-3 is more significant in T cell activation, proliferation and IFN-γ secretion than blocking the PD-1 pathway [155]. Under tolerance conditions, signal transduction through the PD-1/PD-L1 and FGL1/LAG-3 pathways have different functional consequences for CD8^+^ T cell subsets; that is, external signaling initiation can affect the tolerance of CD8^+^ T cell subsets through the LAG3 and PD-1 pathways [164]. The most direct manifestation upstream of these consequences is the production of three CD8^+^ T cell subsets with inconsistent expression and localization of LAG3 and PD-1. Further cytolytic functional analysis showed that the T cell subsets with high expression of LAG3 produced higher levels of cytokines, especially IFN-γ, TNF-α and CD107, than those with high expression of PD-1 but were not associated with the expression of the costimulatory molecules inducible T cell co-stimulator (ICOS) and 4-1BB (CD137) [164, 167–169]. The differences in cytokine production patterns mentioned above indicate the independence of the two pathways. In addition, in animal experiments, more than 30% of MC38 tumor-bearing mice were found to have no tumor formation within 150 days of treatment with anti-FGL1/LAG3 as a monotherapy or in combination with anti-PD-1/PD-L1 therapy [50]. Because of the high affinity between FGL1 and LAG3, FGL1/LAG3 and PD-1/PD-L1 can regulate T cells independently, and blocking both these checkpoints can produce synergistic antitumor effects. However, the upregulation of LAG-3 expression in NSCLC is associated with insensitivity to PD-1 axis blockade and a poor prognosis [170], which suggests the independence of immune escape pathways and the potential of the synergistic action of anti-PD-1 and anti-LAG3 antibodies in clinical trials.

## Clinical prospects of anti-FGL1 in cancer therapy

### Anti-FGL1 may overcome immune checkpoint blockade resistance via a tumor-intrinsic mechanism

Based on the above evidence that the FGL1/LAG3 pathway plays an important role in immune evasion during cancer development, anti-FGL1 may help overcome cancer immunotherapeutic resistance as a promising novel checkpoint. Characteristics that are necessary for the immune checkpoint inhibitor (ICI) response include IFN-γ signaling, antigen-presenting signaling, immune-evasive oncogenic signaling, and a mutational signaling landscape [171, 172]. It is well known that the T cell receptor (TCR) on the tumor-reactive T cell surface recognizes tumor neoantigens and subsequently releases IFN-γ, which binds to the IFN-γ receptor (IFN-γR1/R2) on the tumor cell surface, activates downstream JAK-STAT signaling pathways and further initiates PD-L1 transcription in tumor cells [171, 173, 174]. The plasma level of IFN-γ was reported to be obviously increased after the injection of CD8^+^OT-1T cells together with the FGL1 antibody 177R4 into syngeneic mice (Table 3), which further proves that the blockade of FGL1 by a monoclonal antibody stimulates T cell immunity in a manner similar to anti-LAG3 therapy [50]. Moreover, FGL1 is highly secreted from tumor cells, and higher plasma levels of FGL1 are associated with resistance to ICIs and poor prognosis in cancer patients [50]. Hence, using FGL1 agents can theoretically enhance the antitumor effect of T cells and thus affect the acquired resistance of ICIs in patients with differential levels of PD-L1 expression.

**Table 3:** Clinical and preclinical data related to anti-FGL1 therapy

Number	Year	Type of tumor	Human/mouse/cell line	Name of FGL1 antibody	Conclusions	References
1	2021	PDAC	Cell lines	FGL1 antibody, (Proteintech)	Anti-FGL1 was associated with lipid metabolism and cell growth in PDAC	[115]
2	2021	HCC	Cell lines	FGL1 antibody, (Proteintech)	Anti-FGL1 eliminated resistance to sorafenib in HCC cells	[116]
3	2020	NSCLC	Cell lines	ab197357 (Abcam)	Anti-FGL1 increased the sensitivity of NSCLC cells to gefitinib	[114]
4	2019	Murine colon adenocarcinoma	Cell lines and mouse model	Anti-mouse FGL1 (clone 177R4)	Both anti-FGL1 and anti-LAG3 mAbs significantly controlled the growth of tumors derived from the MC38 murine colon that were inoculated into syngeneic C57BL/6 mice	[50]
5	2019	Murine liver cell	Cell lines and mouse model		Both anti-FGL1 and anti-LAG3 mAbs significantly controlled the growth of tumors derived from established Hepa1-6 murine liver cell lines that were inoculated into syngeneic C57BL/6 mice	

HCC: hepatocellular carcinoma; NSCLC: non-small cell lung cancer; PDAC: pancreatic ductal adenocarcinoma

Furthermore, antigen presentation blockade is another intrinsic factor underlying ICI resistance, which is mainly caused by MHC deficiency and loss of tumor antigenicity [175, 176]. MHC II is the first identified ligand of LAG3 [177]. However, their binding does not affect antitumor activity, suggesting that MHC II is not solely responsible for the function of LAG3. More valuable ligands presumably restart the “immune brake” by binding with LAG3. Therefore, as a newly discovered high-affinity ligand of LAG3, FGL1 has the potential to induce ICI resistance in a receptor-ligand interdependent manner [50]. Moreover, cancers with a high tumor mutation burden (TMB) and high neoantigen expression, such as melanoma and NSCLC, are generally more sensitive to immune checkpoint blockade [60, 173, 178]. However, these patients were observed to have higher plasma levels of FGL1 expression after the acquisition of ICI resistance [50]. Thus, the FGL1 secretion level in plasma is a potential biomarker for identifying patients who will not benefit from ICI treatment. Finally, Wnt/β-catenin is an essential signaling pathway in cancer development [179, 180]. Emerging evidence indicates that the Wnt5α-β-catenin-PPARγ signaling pathway drives a metabolic program that triggers dendritic cell (DC) tolerance and immunotherapy resistance [181, 182]. PPARγ is the ultimate target of FGL1-induced lipid synthesis in adipocytes, suggesting that FGL1 has the potential to induce immunosuppression by regulating PPARγ synthesis in metabolic programs [56].

### Anti-FGL1 may overcome immune checkpoint blockade resistance via exogenous factors

**Fig. 5:**
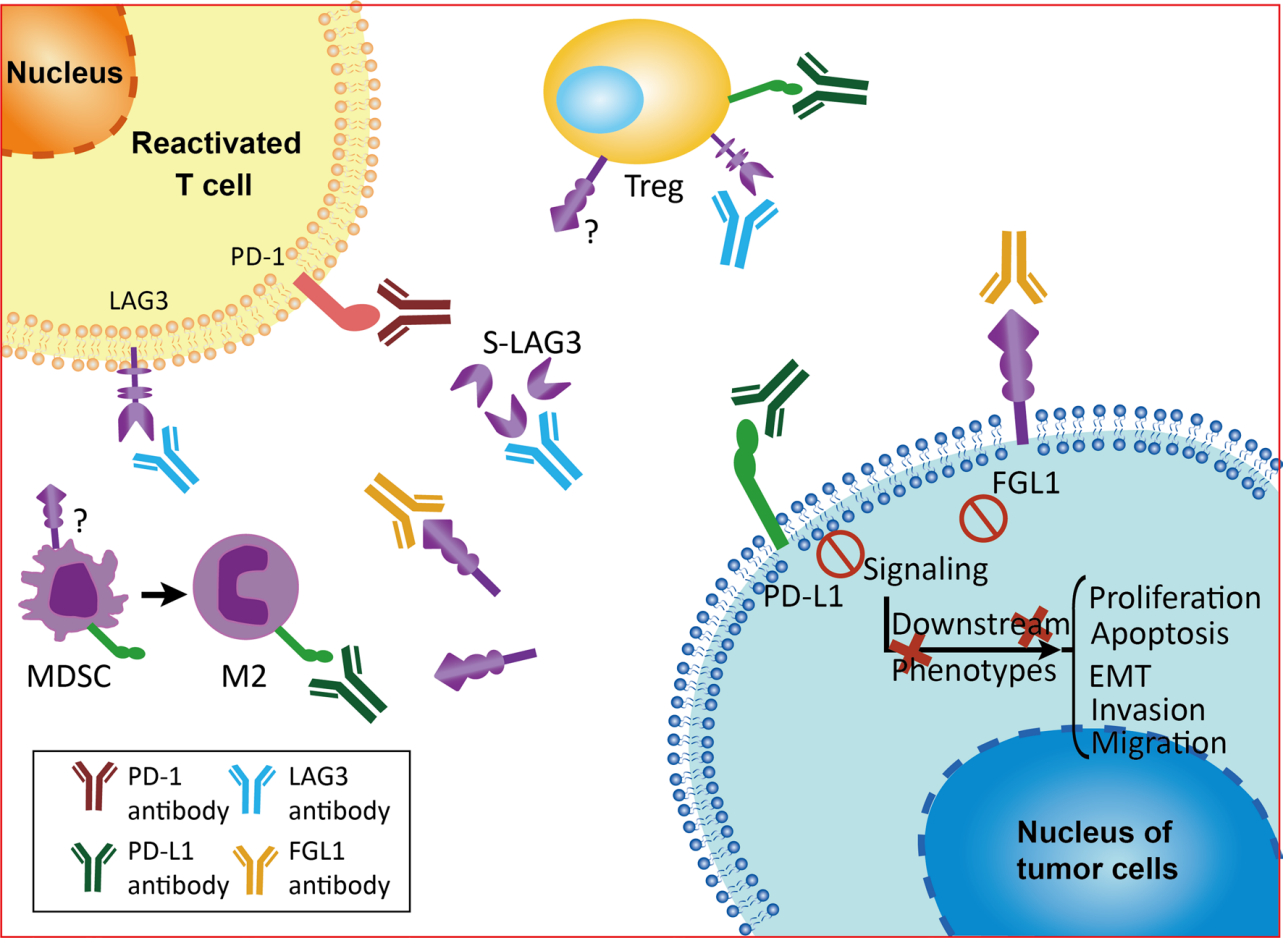
Combined use of antibodies targeting PD-1/PD-L1 and FGL1/LAG3 in the tumor microenvironment. The PD-1/P-L1 and FGL1/LAG3 signaling pathways can be inhibited by the corresponding antibodies, leading to the reactivation of nonactive T cells, while downstream pathways and phenotypes associated with tumor cell progression are weakened. In addition to TILs, MDSCs, TAMs (M2) and Tregs in the TME are known to mediate antitumor immunity through the PD-1/PD-L1 pathway, but their roles in the FGL1/LAG3 pathway remain to be determined

In addition to intrinsic factors, alterations in the functions and numbers of immune effector cells (mainly CD8^+^ T cells) in the tumor microenvironment (TME) are challenging for immunotherapies [183]. Various immune cells (DCs, Tregs, myeloid-derived suppressor cells (MDSCs)) and their related secretory products and metabolites in the TME play essential roles in tumor development, metastasis and immunotherapeutic response [184]. Persistent antigen exposure in the TME clearly results in sustained LAG3 expression, thereby having a substantial effect on the immunosuppression status and cytokine production. As a major LAG3 functional ligand independent of MHC-II, FGL1 is positively correlated with MDSC and Treg populations and negatively correlated with CD8^+^ T cells as determined by analysis of the TIMER database (http://timer.cistrome.org/). The regulatory effect of FGL1 on the TME was further demonstrated in a breast cancer model constructed using biomimetic nanomaterials designed to deliver a short interfering RNA targeting FGL1 [185]. Moreover, FGL1 was shown to be closely associated with tumor cell invasion and metastasis via acquisition of the EMT phenotype, which was underlined by bidirectional crosstalk between tumor cells and the surrounding TME [93, 94]. This evidence indicates preferential suppression of T cell immunity in the TME upon FGL1-LAG3 signaling. Indeed, the current insights into the mechanism by which FGL1 induces immunotherapy resistance via immunosuppression of the TME are relatively limited, and more in-depth investigations are still needed (Fig. 5).

### Potential toxicity patterns of anti-FGL1 therapy

Based on clinical trials and medical practices, ICI therapies not only initiate the activation of an anticancer immune response but also cause a wide range of immune-related adverse events (irAEs), and the costs of immunotherapies continue to increase due to the diversity of T cell expansion and cell infiltration [186]. Different from the toxic effects of traditional chemotherapy, the most frequently affected organs are the skin, digestive system (gastrointestinal, liver) and endocrine system, with symptoms of nausea, diarrhea, pruritus, rashes and thyroid problems, and fatal irAEs include myocarditis, pneumonia, hepatitis and meningitis [187]. The frequency of irAEs and potential toxicity patterns are dependent on the immunotherapy strategy, exposure time, medicinal dose administered and individual intrinsic risk factors of the patient [186].

Some novel emerging immune checkpoints are approved for clinical trials in combination with CTLA-4 or PD-1(L1) blockade, such as anti-LAG-3, which increases the probability and new descriptions of irAEs (Table 4). Multiple new agents have been identified as targeting LAG3 across various tumors [188]. The most common agent-related AEs, fatigue and nausea, are well tolerated. Nonetheless, the discovery of more novel checkpoints is still urgently needed in the paradigm of cancer treatment. Among the potential candidates, FGL1 has promise despite that it remains in the preclinical stage, and researchers are awaiting more advanced data. Theoretically, as another ligand of LAG3 in addition to the canonical ligand MHC-II, FGL1 may have more advantages than anti-LAG3 in regards to irAEs. Future studies should also optimize the selection of new ICIs by screening potential predictive biomarkers for the irAE risks [189].

**Table 4:** Summary of clinical trials utilizing agents targeting LAG3 alone or in combination with others

Drug	Clinical trial number	Phase	Status	Tumor type	Clinical efficacy	Safety	Details
MGD013 (tebotelimab)	NCT04212221	I/II	Recruiting	HCC	NA	NA	In combination with brivanib alaninate (ZL-2301)
	NCT04653038	I	Recruiting	Melanoma	NA	NA	Single agent
	NCT04634825	II	Recruiting	HNSCC	NA	NA	Tebotelimab or retifanlimab (PD-1 antibody) plus enoblituzumab (B7-H3 antibody)
	NCT03219268		Active, not recruiting	Neoplasms (unresectable or metastatic)	NA	Fatigue: 19% Nausea: 11% Grade ≥ 3 TRAEs: 23.2%	irAEs were consistent with events observed in anti-PD-1 antibodies
BMS-986016 (relatlimab)	NCT03470922	II/III	Active, not recruiting	Melanoma (unresectable or metastatic)	NA	NA	In combination with nivolumab (anti-PD-1 mAb)
REGN3767 (fianlimab)	NCT03005782	I	Recruiting	Malignancies	ORR: 64% (21 of 33 patients; 3 complete responses, 18 partial responses)	***Monotherapy TRAE*** Nausea: 22.2% Increased ALT and AST: 3.7% ***Combination therapy TRAE*** Fatigue: 31% Rash: 23% Grade ≥ 3 irAEs: Hypothyroidism: 2.4%	In combination with Cemiplimab (REGN2801)
BI 754111	NCT03156114 NCT03433898 NCT03697304 NCT03780725	I/II	Active, not recruiting/ Recruiting/ completed	Carcinoma, NSCLC, Head and neck neoplasms	NA	Any AEs: 86.7%; Any irAEs: 21.1% IRRs: 4.9% Hypothyroidism: 3.2%	In combination with 754091 (anti-PD-1 mAb)
IMP321 (eftilagimod alpha)	NCT02676869	I	Completed	Stage IV/III melanoma	ORR: 33–50%	No dose-limiting toxicities Main AE: IRR	In combination with pembrolizumab (anti-PD-1 mAb)
	NCT03625323	II	Recruiting	NSCLC HNSCC	NA	NA	
BMS-986213 (relatlimab, nivolumab)	NCT03662659	II	Active, not recruiting	GC/GEJC	NA	NA	Relatlimab/nivolumab, given in combination with chemotherapy
BMS-986016 (relatlimab)	NCT03607890	II	Recruiting	MSI-H tumors	NA	NA	Utilized in patients with MSI-H solid tumors refractory to prior PD-(L)1 therapy
LAG525 (IMP701)	NCT03365791	II	Completed	Advanced solid and hematologic malignancies	NA	AEs: 98.7% Fatigue: 36.84% Nausea: 34.21% SAEs: 42.1% Pneumonia: 6.58%	In combination with spartalizumab (PDR001) (anti-PD-1 mAb)
Sym022	NCT0331141	I	Recruiting	Metastatic cancer solid tumors lymphoma	NA	Grade ≥ 3 TRAEs: Increased CPK: 10% Decreased lymphocytes: 5% Hypophysitis: 5%	In combination with Sym021 (anti-PD-1) or Sym023 (anti-TIM3)
LBL-007	NCT04640545	I	Recruiting	Advanced melanoma	NA	NA	In combination with toripalimab (anti-PD-1 mAb)
FS0118	NCT03440437	I/II	Recruiting	Advanced malignancies	NA	NA	Single agent
TSR-033	NCT03250832	I	Active, not recruiting	Neoplasms	NA	NA	TSR-033/dostarlimab (anti-PD-1 mAb) in combination with chemotherapy

ALT: alanine aminotransferases; AST: aspartate aminotransferases; MSI-H: high levels of microsatellite instability; GEJC: esophagogastric junction cancer; HNSCC: head and neck squamous cell carcinoma; irAE: immune-related adverse event; IRR: infusion-related reaction; NA: not available; ORR: objective response rate; TRAE: treatment-related adverse event; SAE: severe adverse event; mAb: monoclonal antibody

### Future directions of anti-FGL1 in cancer therapy

Despite the early stage of tumor research, FGL1 is a new immune checkpoint molecule that is thought to have a promising future in clinical applications, especially in NSCLC immunotherapy [190–193], which is attributed to its overexpression in NSCLC cells and close correlations with immune regulation, tumor neovascularization [194], EMT progression [195], resistance and metastasis [116, 196, 197]. The novel discovery of a high-affinity interaction between FGL1 and LAG3 in immunology marks a major breakthrough in research on immune checkpoint blockade therapy. This strategy holds great potential as a third-generation immune checkpoint blockade after targeting CTLA-4 and PD-1/PD-L1 [198]. Currently, multiple clinical trials evaluating the synergistic effects of anti-LAG3 and anti-PD-1 antibodies are ongoing, and significant efficacy has been achieved in completed trials on melanoma (stage III/IV), metastatic breast cancer and RCC (stage IV) [163, 165]. Although the number of ongoing clinical trials targeting FGL1 are not sufficient, a significant positive correlation between FGL1 expression and long-term prognosis has been observed for patients with multiple types of metastatic cancer who are treated with anti-PD-1/PD-L1 therapy (Table 2)*.* Based on the above results, patients with advanced-stage tumors reliant on ICIs are likely to benefit from anti-FGL1 therapy. More prospective clinical trials targeting FGL1 alone or in combination with other ICI agents are needed to facilitate translocation from bench to bedside.

## Conclusions

As an acute inflammatory factor secreted by the liver, FGL1 is upregulated in various solid tumors and associated with the EMT, proliferation, apoptosis and drug resistance of tumors as well as with poor prognosis. Most importantly, an immunosuppressive pathway distinct from that of PD-1/PD-L1 forms between FGL1 and the inhibitory receptor LAG3. Targeting FGL1 acts on both tumor cells themselves and immune cells, which exerts a two-way synergistic effect on advanced cancers through a targeted therapeutic strategy. Therefore, FGL1 demonstrates significant advantages as a biomarker for predicting the efficacy of anti-PD-1/PD-L1 therapy in patients with advanced tumors, and targeting FGL1 has promise as another promising immune checkpoint blockade strategy in clinical trials. Novel therapeutic strategies targeting FGL1 should be further explored in the treatment of cancer.

## Data Availability

Data sharing not applicable to this article as no datasets were generated or analyzed during the current study.

## Abbreviations

AP-1: Activating protein-1
CD107/137/ 163/223/274: Cluster of Differentiation 107/137/163/223/274
C/EBPβ: CCAAT/enhancer-binding protein β
CTC: Circulating tumor cell
DC: Dendritic cell
EGFR: Epidermal growth factor receptor
ERK: Extracellular regulated protein kinases
FGL1: Fibrinogen-like protein 1
HNF-1α: Hepatocyte nuclear factor-1α
ICOS: Inducible T cell co-stimulator
irRC: Immune-related response criteria
IL-6/12: Interleukin-6/12
IFN-γ: Interferon-γ
JNK: Jun N-terminal kinase
LKB1: Liver kinase B1
LAG3: Lymphocyte activation gene 3
MMP: Matrix metalloprotease
mAb: Monoclonal antibody
mTOR: Mammalian target of rapamycin
NK: Natural killer
NRF2: Nuclear factor erythroid-2-related factor 2
NSCLC: Non-small cell lung cancer
PD-1: Programmed cell death protein 1
pDC: Plasmacytoid dendritic cell
PD-L1: Programmed cell death ligand 1
PPARγ: Peroxisome proliferator-activated receptor gamma
RA: Rheumatoid arthritis
SOD1: Superoxide dismutase 1
STAT3: Signal transducer and activator of transcription 3
TCGA: The cancer genome atlas
TCR: T cell receptor
TGF-β: Transforming growth factor-β
TIGIT: T cell immunoreceptor with Ig and ITIM domains
TIL: Tumor-infiltrating lymphocyte
TIM3: T cell immunoglobulin domain and mucin domain-3
TKI: Tyrosine kinase inhibitor
TMB: Tumor mutation burden
TNF-α: Tumor necrosis factor-α
VISTA: V-domain Ig-containing Suppressor of T cell Activation
ZO-1: Zonula occludens-1
